# Position-Dependent Influence of the Three Trp Residues on the Membrane Activity of the Antimicrobial Peptide, Tritrpticin

**DOI:** 10.3390/antibiotics3040595

**Published:** 2014-11-06

**Authors:** Mauricio Arias, Leonard T. Nguyen, Andrea M. Kuczynski, Tore Lejon, Hans J. Vogel

**Affiliations:** 1 Biochemistry Research Group, Department of Biological Sciences, University of Calgary, 2500 University Dr. NW, Calgary, AB T2N 1N4, Canada; E-Mails: ariasm@ucalgary.ca (M.A.); lltnguye@ucalgary.ca (L.T.N.); amkuczyn@ucalgary.ca (A.M.K.); 2 Department of Chemistry, Faculty of Science, UiT—The Artic University of Norway, Tromsø N-9037, Norway; E-Mail: tore.lejon@uit.no

**Keywords:** antimicrobial peptides, tritrpticin, membrane permeabilization, tryptophan, fluorescence spectroscopy, NMR spectroscopy

## Abstract

Antimicrobial peptides (AMPs) constitute promising candidates for the development of new antibiotics. Among the ever-expanding family of AMPs, tritrpticin has strong antimicrobial activity against a broad range of pathogens. This 13-residue peptide has an unusual amino acid sequence that is almost symmetrical and features three central Trp residues with two Arg residues near each end of the peptide. In this work, the role of the three sequential Trp residues in tritrpticin was studied in a systematic fashion by making a series of synthetic peptides with single-, double- and triple-Trp substitutions to Tyr or Ala. ^1^H NMR and fluorescence spectroscopy demonstrated the ability of all of the tritrpticin-analog peptides to interact with negatively-charged membranes. Consequently, most tritrpticin analogs exhibited the ability to permeabilize synthetic ePC:ePG (egg-yolk phosphatidylcholine (ePC), egg-yolk phosphatidylglycerol (ePG)) vesicles and live *Escherichia coli* bacteria. The membrane perturbation characteristics were highly dependent on the location of the Trp residue substitution, with Trp6 being the most important residue and Trp8 the least. The membrane permeabilization activity of the peptides in synthetic and biological membranes was directly correlated with the antimicrobial potency of the peptides against *E. coli*. These results contribute to the understanding of the role of each of the three Trp residues to the antimicrobial activity of tritrpticin.

## 1. Introduction

The growing number of bacterial strains that are resistant to currently used antibiotics has been recognized as a major threat to public health by the World Health Organization [1]. The inefficacy of current antibiotics has triggered a crucial search for new sources and types of antimicrobial agents. One potential source for novel antibiotics could be found among the many antimicrobial peptides (AMPs) [2,3,4,5]. AMPs have been intensively studied for the last three decades, as they constitute a crucial element of the innate immune system of higher organisms [6,7,8], highlighting their value as potential therapeutic agents. Additionally, AMPs have been found in virtually every living organism on Earth, from bacteria to humans [8,9]. Their ability to efficiently kill microbial invaders by a direct mechanism or by modulation of the host immune systems represents a novel mode of action compared to currently used therapeutic agents against infections [10,11,12]. A multidisciplinary approach in the study and discovery of AMPs has already resulted in databases containing the amino acid sequences of more than 5000 antimicrobial peptides, encompassing naturally-derived peptides sequences, as well as peptide analogs and predicted sequences [13,14]. Despite all of the efforts studying these peptides, the mechanism of action of many AMPs is not yet completely understood.

Tritrpticin is a relatively short 13-amino acid residue peptide that is part of the cathelicidin family of AMPs. Originally, it was discovered in porcine neutrophils and was shown to exhibit a strong antimicrobial activity against Gram-positive and Gram-negative bacteria, as well as fungi [15]. Due to its amino acid composition, containing three Trp and four Arg residues, it is also a member of the Arg/Trp-rich family of AMPs [16]. In a previous study, we already investigated the effects of the substitution of the Arg residues with various natural and unnatural amino acids on the antimicrobial activity [17]. Here, we will focus on the Trp residues, because these have been identified as key elements for several Trp-rich AMPs in terms of regulating their antimicrobial activities and mechanism of action [18,19,20,21,22]. Substitutions of all three Trp residues simultaneously in tritrpticin with Ala, Phe and Tyr have already been reported. Mutation of all Trp residues to Phe resulted in a more potent antimicrobial peptide, while substitution to Tyr and Ala had a negative effect on tritrpticin’s antimicrobial activity. The preferential localization of the Trp indole rings in the water-lipid interface when these peptides are bound to the membrane appears to be directly related to the potent activity of tritrpticin and related cationic Trp-rich AMPs [21,22,23]. Interestingly, substitutions of Trp with the more polar hydroxy-Trp did not alter the antimicrobial activity, although it caused changes in the mode of action of the peptide [24].

While tritrpticin has been extensively studied by making complete amino acid substitutions of all three Trp residues simultaneously, a systematic study of the importance of each individual Trp residue, from the central Trp-cluster in tritrpticin, has not yet been carried out. In this study, the *C*-terminally amidated version of tritrpticin, Tritrp1, was used as the reference. Tritrp1 exhibits a slightly higher antimicrobial activity in comparison to its precursor [17,22]. Although *C*-terminal amidation of tritrpticin has not been established *in vivo*, the related Trp/Arg-rich peptide, indolicidin, is naturally *C*-terminally amidated [25]. Synthetic peptides with single, double and triple substitutions of the Trp residues in Tritrp1 were prepared using Tyr and Ala. The Tyr substitutions were included in this work to preserve the preference of the amino acid side chain for localization in the membrane interface [26,27]. On the other hand, Ala substitution would completely remove the side chains that contribute to preferential membrane interface binding. Our results show that the three Trp residues of Tritrp1 exert a position-dependent influence on the antimicrobial activity. This effect could be correlated with the altered *in vitro* and *in vivo* membrane permeabilization properties of the peptides in this study.

## 2. Results and Discussion

### 2.1. Peptide Design

The amino acid sequences of the peptides that were used in this study are shown in Table 1. Tyr or Ala residues were used in a systematic manner to substitute for the three Trp residues to introduce moderate or major changes, respectively, compared to the Tritrp1 parent peptide.

**Table 1:** The amino acid sequences and antimicrobial activities reported as minimal inhibitory (MIC) and bactericidal concentrations (MBC) for Tritrp1 and its analogs against *E. coli* ATCC 25922.

Peptide	Sequence	MIC (µM)	MBC (µM)
Tritrp1	VRRFPWWWPFLRR-NH_2_	4	4
**Trp-to-Tyr analogs**			
W6Y	VRRFP**Y**WWPFLRR-NH_2_	16	16
W7Y	VRRFPW**Y**WPFLRR-NH_2_	8	8
W8Y	VRRFPWW**Y**PFLRR-NH_2_	4	4
W67Y	VRRFP**YY**WPFLRR-NH_2_	32	32
W78Y	VRRFPW**YY**PFLRR-NH_2_	16	16
W68Y	VRRFP**Y**W**Y**PFLRR-NH_2_	16	16
Y-Tritrp	VRRFP**YYY**PFLRR-NH_2_	16–32	16–32
**Trp-to-Ala analogs**			
W6A	VRRFP**A**WWPFLRR-NH_2_	32–64	32–64
W7A	VRRFPW**A**WPFLRR-NH_2_	16	16
W8A	VRRFPWW**A**PFLRR-NH_2_	8	8
W67A	VRRFP**AA**WPFLRR-NH_2_	64–128	64–128
W78A	VRRFPW**AA**PFLRR-NH_2_	64–128	64–128
W68A	VRRFP**A**W**A**PFLRR-NH_2_	64–128	64–128
A-Tritrp	VRRFP**AAA**PFLRR-NH_2_	>128	>128

### 2.2. Antibacterial Activity

The antimicrobial activities of all of the peptides are shown in Table 1. Many of the peptides lost some activity against *E. coli* ATCC25922 compared to the parent peptide, with the triple-Trp-to-Ala substitution rendering the peptide completely inactive in the concentration range tested. The loss of antimicrobial activity for A-Tritrp was also observed in studies involving other bacterial strains [21]. For the remaining peptides, the antimicrobial activity was bactericidal rather than bacteriostatic, as indicated by the matching values for the MICs and minimal bactericidal concentrations (MBCs).

The Tyr-derived peptides in general exhibited higher antimicrobial activities than their Ala-derived counterparts. These results indicate that at the center of Tritrp1, where the Trp residues are located, a certain degree of polarity and aromaticity is preferred, but not mandatory in order to exhibit antimicrobial activity. However, a clear pattern emerges when considering the single-substituted peptides. The Trp located at position 6 (Trp6) appears to be very important for the antimicrobial activity. Substitution of this residue by either Tyr or Ala induced a substantially higher loss of antimicrobial activity. In comparison, the substitution of Trp8 with Tyr did not affect the activity of the peptide, while the substitution of Trp7 plays an intermediate role for activity.

Double substitutions with Ala residues reduced the antimicrobial activity of the peptides considerably, with no apparent difference between the positions of the substituted Trp residues. Nevertheless, the double-Trp-to-Tyr-substituted peptides’ activities enforce the notion of a dominant role for Trp6 and Trp7 for the antimicrobial activity, with W67Y being less antimicrobial than W68Y and W78Y. Interestingly, triple substitutions with Tyr reduced the bactericidal potency of the peptide, similarly as described by Schibli *et al.* [22]. However, this potency was close to the potency of the W67Y peptide. Likewise, several other antimicrobial peptides have been reported to lose bactericidal activity due to Trp-to-Tyr mutations, e.g., indolicidin [28], synthetic hexapeptides [29], as well as lactoferricin- and lysozyme-derived peptides [30,31].

Previous work established that the substitution of all three Trp residues with Phe increased the antimicrobial activity of Tritrp1 [21,22]. Since Phe, unlike Trp and Tyr, does not have a preference for the membrane interface [32,33], this effect was thought to be related to the increased hydrophobic character of the peptide. These results reinforce the importance of the hydrophobic and/or aromatic character of the residues located at the core of the peptide.

### 2.3. ^1^H NMR Spectroscopy

**Figure 1:**
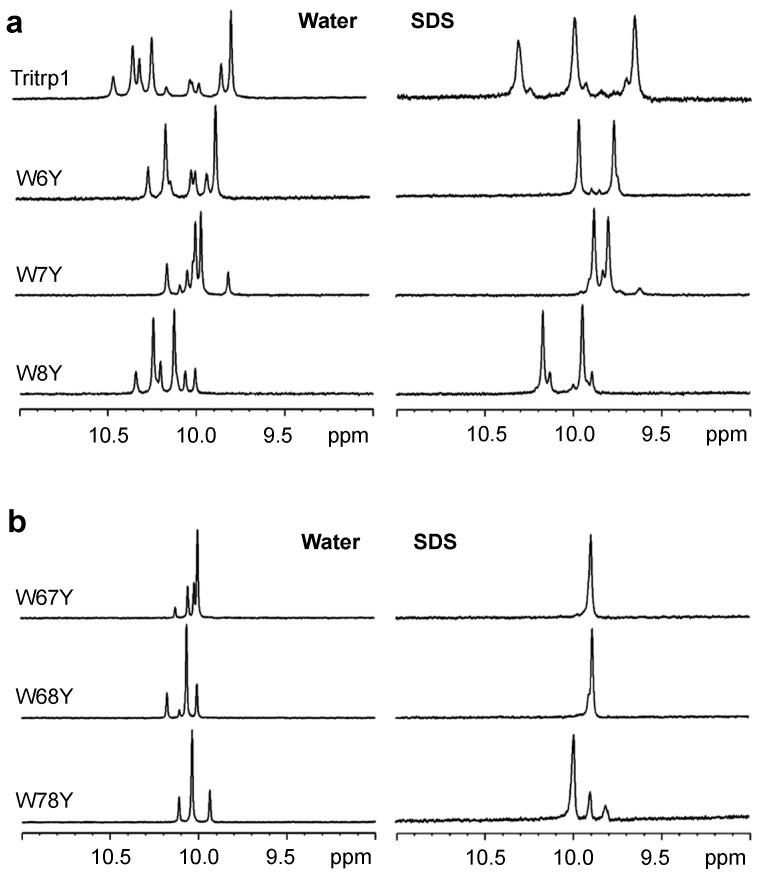
One-dimensional proton NMR spectra recorded for the Hε1-tryptophan region of single- (**a**) and double- (**b**) Trp-substituted Tritrp1 peptides with Tyr in aqueous solution (**left**) and in the presence of d_25_-SDS micelles (**right**) at 37 °C.

**Figure 2:**
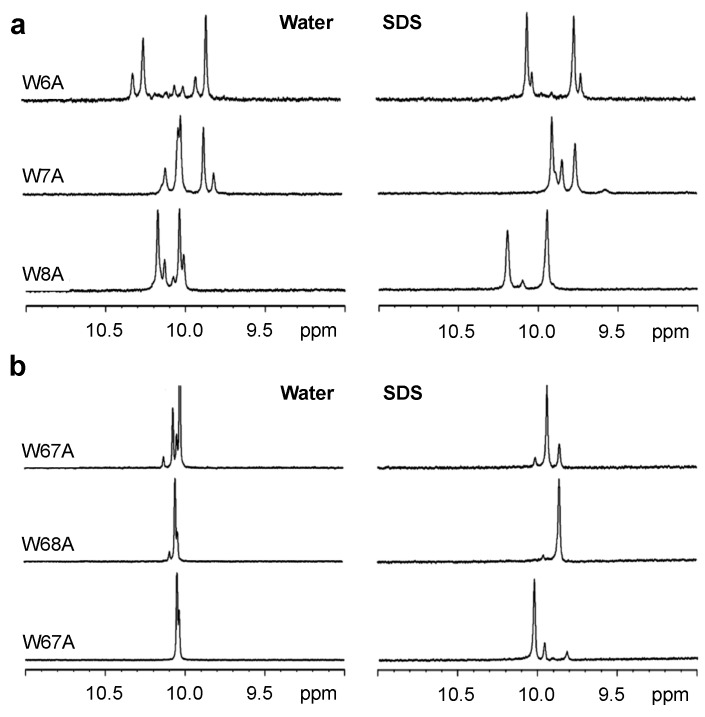
One-dimensional proton NMR spectra recorded for the Hε1-tryptophan region of single- (**a**) and double- (**b**) Trp-substituted Tritrp1 peptides with Ala in aqueous solution (**left**) and in the presence of d_25_-SDS micelles (**right**) at 37 °C.

The binding of linear AMPs to lipid bilayers is normally coupled with changes in the conformation of the peptides. In many cases, most peptides do not adopt a single conformation in buffer, but they usually do so when bound to a membrane or membrane-mimetic surface [34]. In the case of Tritrp1, it has been shown that the two Pro residues flanking the Trp residues are responsible for a high conformational heterogeneity, which is caused by *cis-trans* isomerization around the X-Pro (with X representing any amino acid residue that precedes the proline residue) bonds in aqueous environments. Upon binding to lipid micelles, the peptide acquired a more rigid and defined conformation, as shown by Schibli *et al.* [22,35]. In order to study the effects of membrane binding on the heterogeneity of the Tyr- and Ala-substituted Tritrp1 peptides, ^1^H NMR spectra were acquired. Each peptide was studied in aqueous solution and in the presence of SDS (sodium dodecyl sulfate) micelles, as depicted in Figure 1 and Figure 2. SDS micelles, while not a perfect membrane mimetic, have been widely used for the NMR structure determination of AMPs in solution [36,37,38]. The size of these micelles allows for regular high resolution solution state ^1^H NMR studies to be performed [39,40]. In addition, the negatively-charged nature of the SDS detergent emulates the negatively-charged surface of the bacterial membrane [36,37,38,41]. The spectral region between 11.0 and 9.0 ppm in the ^1^H NMR spectra exclusively shows the resonances corresponding to the Hε1 protons of the Trp indole rings, as shown in Figure 1 and Figure 2. This region is far removed from the highly crowded upfield regions of the spectra, allowing a direct interpretation of the conformational status of the Trp residues in the peptide samples.

In aqueous solution, all of the single-substituted peptides (Figure 1a and Figure 2a) were characterized by the presence of two strong and several weaker resonances. This reflects the existence of different conformers in aqueous solution. The *cis-trans* isomerization of the two X-Pro bonds in all of our peptides is likely responsible for the multiple conformation of the peptides, as was previously described for Tritrp1 [22]. Upon binding to the SDS micelles, the number of Hε1 resonances is significantly reduced. In most cases, only two strong peaks are observed, although in some cases, a weaker pair of resonances is still detected. These spectra indicate that all of the single-substituted peptides interacting with the micelles acquired a main and stable conformation. However a minor conformation was still present, although it represents only a small fraction of the peptides.

For the double-substituted peptides, a similar pattern was observed (Figure 1b and Figure 2b). Due to the presence of only one Trp residue in the peptides, one strong Hε1 peak was expected. The transition from the aqueous environment to SDS micelles was again accompanied by a considerable reduction in the number of peaks, again indicating a transition from several conformers in the aqueous environment to only one major micelle-bound conformer (Figure 1b and Figure 2b).

### 2.4. Tryptophan Fluorescence Spectroscopy

The fluorescence emission of the Trp indole side-chain is highly sensitive to the polarity of its environment, which allows for the study of the interaction between Trp-containing peptides and lipid bilayers. It is expected that upon binding of the peptides to membrane mimetic surfaces, the hydrophobicity of the environment surrounding the Trp residues would increase, leading to a shift of the maximum emission wavelengths to lower values, commonly referred to as the blue shift [42]. The blue shifts induced upon the interaction of Tritrp1 and its Trp-substituted peptides with ePC:ePG (egg-yolk phosphatidylcholine (ePC), egg-yolk phosphatidylglycerol (ePG)) and ePC:Chol (egg-yolk phosphatidylcholine (ePC), cholesterol (Chol)) vesicles are depicted in Table 2. The use of a zwitterionic phospholipid (PC) and cholesterol simulated the electrically neutral and fluidity/rigidity characteristics of a eukaryotic membrane [22,43], while the negatively-charged phospholipid (PG) with PC can be used to emulate the negatively-charged surface of bacterial cell membranes, as previously indicated [22].

A substantial blue shift of close to 20 nm is observed for all peptides upon binding to ePC:ePG large unilamellar vesicles (LUVs). There is no significant trend in the blue shifts of the peptides in the presence of these vesicles; however, these shifts are all higher than that observed for Tritrp1 (14 nm). This can be partially explained by the lower maximum emission wavelength of Tritrp1 with ePC:ePG in buffer (351 nm) compared to 353–356 nm for the other peptides. This suggests that, already in aqueous solution, the presence of all three Trp residues simultaneously provides a slightly hydrophobic environment that influences its fluorescence emission maximum. In contrast, no considerable blue shifts were observed with ePC:Chol vesicles, suggesting that the environment of the Trp residues was not considerably changed in the presence of zwitterionic membranes.

**Table 2:** Maximum emission wavelengths (λ_max_) in buffer and blue shifts (nm) of the Trp-substituted Tritrp1 analogs upon binding to large unilamellar vesicles (LUVs). ePC, egg-yolk phosphatidylcholine; ePG, egg-yolk phosphatidylglycerol; and Chol, cholesterol.

Peptide	λ_max_	Blue Shift (nm)	
	Buffer	ePC:ePG	ePC:Chol
Tritrp1	351	14	2
W6Y	353	18	1
W7Y	354	17	−1
W8Y	354	18	0
W67Y	354	22	1
W78Y	353	18	0
W68Y	353	23	2
W6A	353	19	0
W7A	356	16	0
W8A	355	20	1
W67A	355	17	0
W78A	354	18	0
W68A	356	16	−2

**Figure 3:**
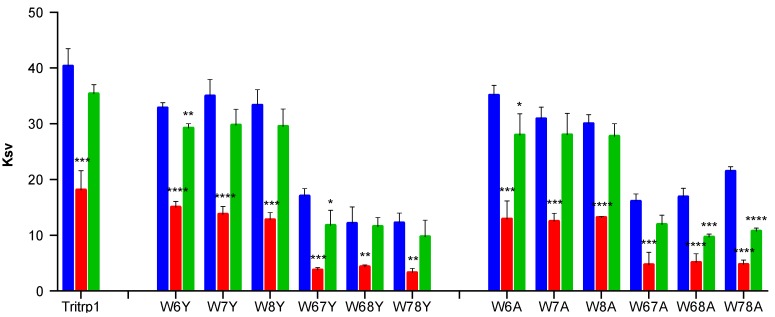
Stern-Volmer constants (Ksv) of the Trp-substituted Tritrp1 analogs as determined by acrylamide quenching experiments in: buffer (blue), ePC:ePG LUVs (red) and ePC:Chol LUVs (green). Results are the mean ± SD (*n* = 3).

As a complement to the blue shift experiments, the peptide-lipid interactions can also be studied by analyzing the solvent accessibility of the Trp residues using a non-ionic fluorescence quencher, such as acrylamide [44,45]. This allows us to estimate the depth of burial of the Trp residues into the membranes. The acrylamide-induced fluorescence quenching characteristics for Tritrp1 and its Trp-substituted analogs in an aqueous environment and upon interaction with LUVs are presented in Figure 3.

A major reduction in the Stern-Volmer constants (Ksv) values is observed for all of the peptides in the presence of negatively-charged ePC:ePG vesicles, indicating that the Trp residues are not exposed to the aqueous solution. In contrast, for most peptides, the Ksv values remain mostly unchanged in the presence of ePC:Chol membranes compared to buffer alone. The Ksv results in the presence of ePC:Chol indicate that the Trp residues are not protected from the acrylamide-induced quenching. In combination with the absence of blue shifts (Figure 3), these results could be consistent with very weak binding or the absence of interactions between the peptides and these zwitterionic model membranes. However, for W68A and W78A, a statistically significant Ksv reduction was observed in the presence of ePC:Chol membranes. Interestingly, these two peptides did not exhibit a considerable blue shift (Table 2).

When comparing the results for all of the Trp-substituted peptides, no clear correlation between the specific Trp mutations and the blue shift or acrylamide quenching results was detected. However, the blue shift and acrylamide quenching results clearly illustrate a more favorable insertion of the Trp residues from the Tritrp1 analogs into the negatively-charged membranes in comparison to zwitterionic membranes.

### 2.5. Calcein Leakage from LUVs

**Figure 4:**
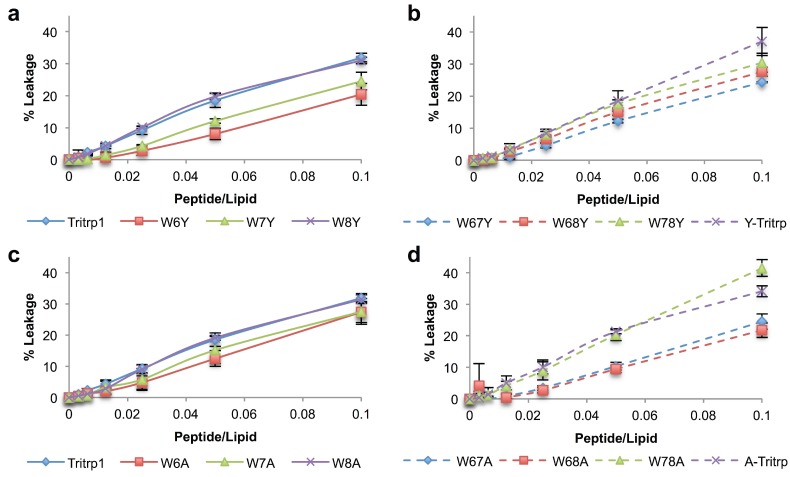
Calcein leakage for Tritrp1 and its analogs in the presence of ePC:ePG (1:1) vesicles. Single-substituted peptides with Tyr (**a**) and Ala (**c**). Double-substituted peptides with Tyr (**b**) and Ala (**d**). Results are the mean ± SD (*n* = 3).

Permeabilization of the bacterial membrane has been identified as one of the possible mechanisms of action for Tritrp1 [22,23,46]. Several studies have established the ability of the peptide to disturb the lipid bilayer and induce leakage from vesicle systems [22,23]. In this work, the permeabilizing ability of the Trp-substituted analogs with ePC:ePG model membrane vesicles was evaluated by measuring the leakage of calcein from these synthetic vesicles of defined composition (Figure 4). It should be noted that the leakage percentages for Tritrp1 reported here are roughly half the value compared to those reported by our group in previous studies [17,22]. This is due to a change in instrumentation to a 96-well plate reader instead of a cuvette-based spectrofluorometer, which utilizes different sample stirring conditions. However, the trends observed amongst the peptides in the two assay systems are the same.

All peptides were able to induce concentration-dependent calcein leakage in negatively-charged vesicles (ePC:ePG). The level of leakage induced by single-substituted peptides is dependent on the position of the Trp residue being replaced (Figure 4a,c). W6Y exhibits the lowest percentage of calcein leakage, indicating that position 6 makes a higher contribution to the permeabilization ability of Tritrp1. In contrast, the peptides with substitutions at Trp8 (W8Y and W8A) retain the same leakage ability as Tritrp1, suggesting that this specific Trp is less important for membrane perturbation. The relevance of Trp7 for membrane permeabilization seems to fall between the Trp residues at positions 6 and 8, as indicated by intermediate levels of leakage for W7Y and W7A.

The results for the double-substituted peptides also support the position-dependent role in membrane permeabilization (Figure 4b,d). Peptides involving Trp6 substitutions (W67Y, W68Y, W67A and W68A) were among the peptides that showed the least amount of leakage, while W78Y and W78A give rise to higher leakage.

**Figure 5:**
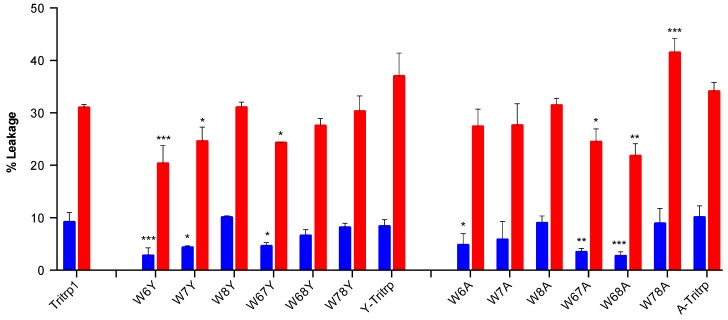
Calcein leakage results for Tritrp1 and its analogs in the presence of ePC:ePG (1:1) vesicles. Two different peptide/lipid (P/L) ratios are depicted as taken from Figure 4, 0.025 (blue) and 0.1 (red). Results are the mean ± SD (*n* = 3).

In order to better compare the influence of different Trp replacements on membrane permeabilization, two peptide-to-lipid ratios (P/L 0.025 and 0.1) were selected, and the results are depicted in Figure 5.

It is clear from Figure 5 that single- and double-Trp substitutions with Tyr induce a position-dependent ability to permeabilize ePC:ePG membranes. The differences among the leakage percentages of W6Y, W7Y and Tritrp1 clearly indicate the importance of these two Trp residues at both P/L ratios (Figure 5). A strong tendency to affect the membrane disturbing ability of the peptides by mutating Trp located at position 6 and 7 can be inferred, with Trp6 being more relevant than Trp7. Mutations of Trp8 did not affect the membrane perturbing ability of the peptides, as detected by the lack of statistically significant difference between the means of W8Y, W8A and Tritrp1. These results correlate very well with the antimicrobial activities described in Table 1, where Trp6 substitutions resulted in reduced antimicrobial activity, and no change in bactericidal activity was observed for W8Y. A similar trend was also observed for the single substitutions with Ala; however, differences among these peptides were within their standard deviations, and only W6A *vs.* Tritrp1 at a low P/L ratio exhibited a statistically significant difference (Figure 5, blue). Nevertheless, for the double-substituted peptides, the results indicate that W67Y, W67A and W68A induced considerably lower leakage than Tritrp1, again illustrating the importance of Trp6 and Trp7. Interestingly, the differences in leakage levels induced by double-Ala-substituted peptides could not be correlated directly with their antimicrobial activities, with MICs of 64–128 µM for W67A, W68A and W78A. This could be related to the broad range of peptide concentrations involved, which is the result of the two-fold dilution setup of the MIC assays.

The peptides with triple substitutions in the Trp residues illustrated an interesting phenomenon, where Y-Tritrp and A-Tritrp were observed to have similar leakage levels as Tritrp1. However, the antimicrobial activities of these two peptides were reduced (Table 1), especially with A-Tritrp having an MIC outside the range of our experiment (>128 µM). For these two peptides, the membrane permeabilization of ePC:ePG vesicles does not seem to correlate with their antimicrobial potency. Previously reported data for Y-Tritrp and a peptide similar to A-Tritrp did not show considerable calcein leakage of negatively-charged liposomes [21,22]. The difference in membrane composition and experimental setup might contribute to the difference observed in leakage behaviour. Lack of direct correlation between calcein leakage and antimicrobial activity has been observed in the past [47], indicating that additional experiments are needed to provide a more accurate description of the mechanism of action of these two Tritrp1 analogs and perhaps other AMPs.

**Figure 6:**
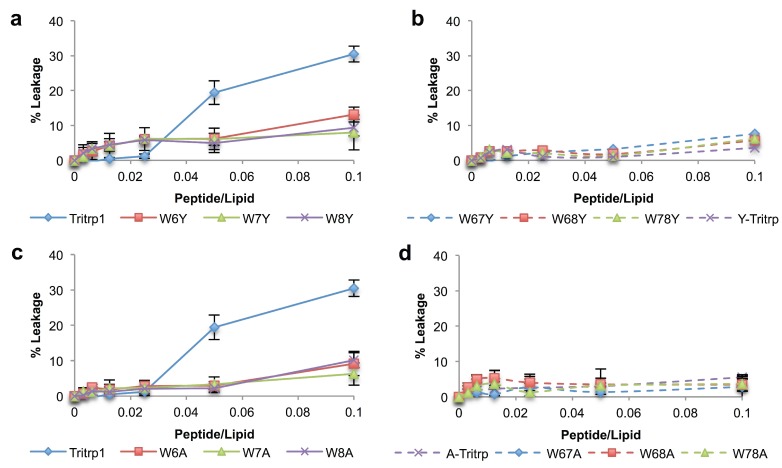
Calcein leakage results for Tritrp1 and its analogs in the presence of ePC:Chol (2.5:1) vesicles. Single-substituted peptides with Tyr (**a**) and Ala (**c**). Double-substituted peptides with Tyr (**b**) and Ala (**d**). Results are the mean ± SD (*n* = 3).

**Figure 7:**
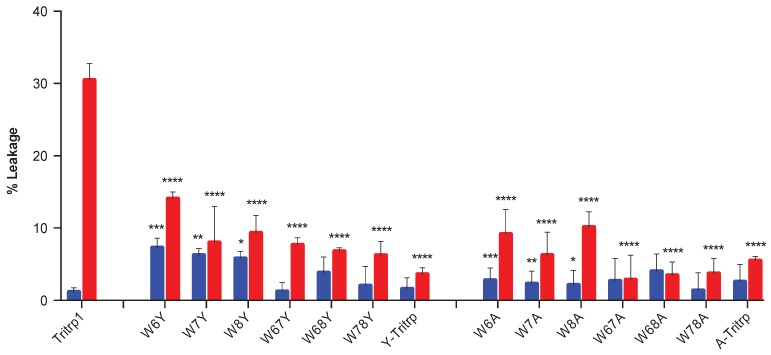
Calcein leakage results for Tritrp1 and its analogs in the presence of ePC:Chol (2.5:1) vesicles. Two different peptide/lipid (P/L) ratios are depicted as taken from Figure 6, 0.025 (blue) and 0.1 (red). Results are the mean ± SD (*n* = 3).

Calcein leakage experiments are normally used to illustrate the importance of the negatively-charged membranes over the zwitterionic membranes. In this work, the preference of Tritrp1 and its analogs to interact with negatively-charged membranes was initially identified by the fluorescence experiments described above (Section 2.4). In order to visualize the permeabilizing ability of the peptides in the presence of a simple eukaryotic model membrane, the calcein leakage experiments were performed using ePC:Chol (2.5:1) vesicles (Figure 6). Additionally, as described for the leakage assays with the ePC:ePG vesicles, two peptide-to-lipid ratios (P/L) were selected, and the results are depicted in Figure 7.

The leakage induced by all of the Tritrp1 analogs with zwitterionic vesicles (Figure 6) was considerably lower than the leakage induced for the negatively-charged vesicles (Figure 4). These results confirm the preference of the peptides for negatively-charged membranes, as previously described for Trp-substituted peptides with Ala and Tyr residues (Table 2 and Figure 3). Interestingly the parent peptide, Tritrp1, also exhibited a similar preference as described by the blue shift (Table 2) and acrylamide quenching experiments (Figure 3). However, large calcein leakage was observed at P/L ratios of 0.05 or higher (Figure 6a,c). This could be an indication that a threshold peptide concentration (P/L > 0.025) is required in order to trigger the permeabilization process in the zwitterionic membranes.

As depicted in Figure 7 for the ePC:Chol membranes, all of the Trp-substituted peptides exhibited a lower ability to induce calcein leakage compared to the parent peptide (Tritrp1), even at high peptide-to-lipid ratios. These results indicate that the substitution of any of the Trp residues for Ala or Tyr in Tritrp1 is sufficient to impede the permeabilization effect on these membranes. The lack of membrane perturbing ability for the Tritrp1 analog peptides is in agreement with the low levels of Trp membrane insertion observed in the blue shift (Table 2) and acrylamide quenching fluorescence studies (Figure 3). Similar results have been described for several AMPs when interacting with cholesterol-containing membranes [48]. The lack of membrane perturbing abilities for these membranes suggests that our analogs might exhibit higher selectivity towards bacterial membranes compared to the original Tritrp1 peptide.

### 2.6. E. coli Inner Membrane Permeabilization

**Figure 8:**
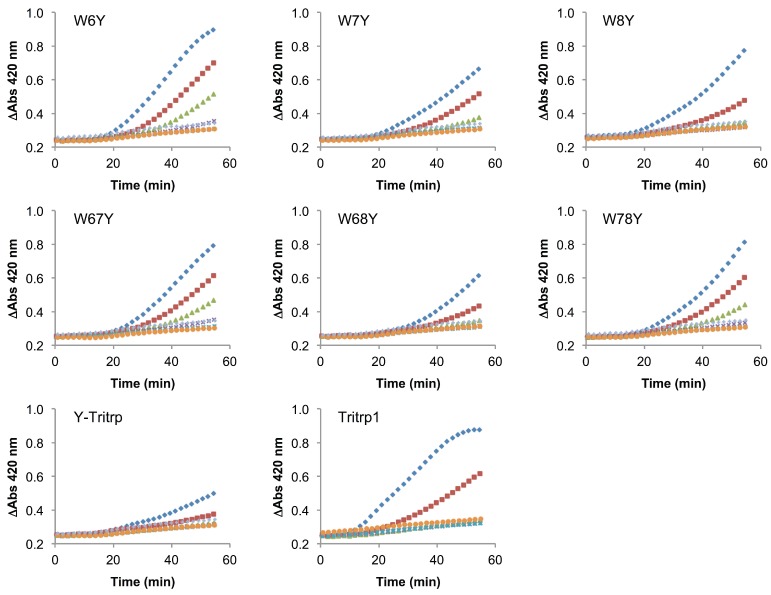
Inner membrane permeabilization induced by Tritrp1 and Trp-to-Tyr-substituted Tritrp1 analogs. Peptide concentrations were MIC (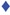), ^1^/_2_ MIC (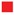), ^1^/_4_ MIC (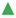), ^1^/_8_ MIC (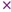), ^1^/_16_ MIC (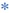), ^1^/_32_ MIC (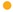) and 0 µM (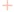). The MIC values for each peptide are derived from Table 1. For peptides with a range of MICs, the higher concentration was used. The results are the average of three independent experiments, and the standard deviation (SD) for two selected peptide concentrations are depicted in Figure 10.

**Figure 9:**
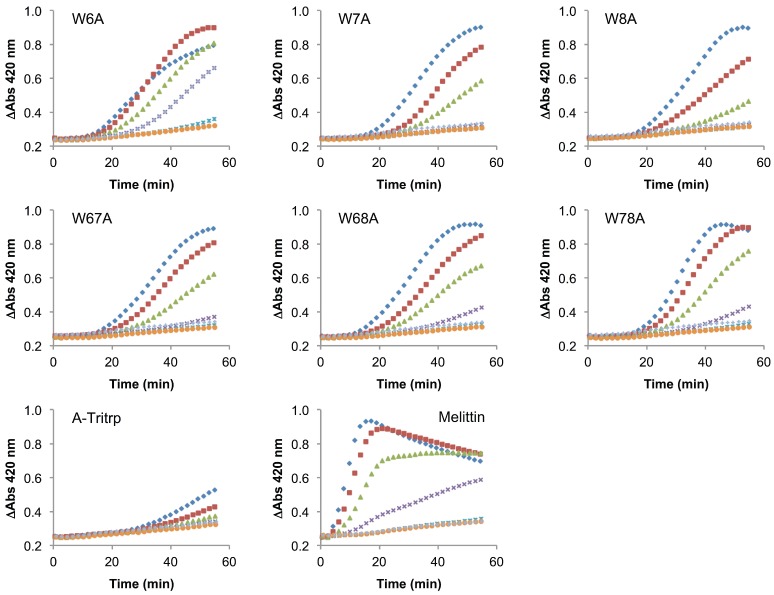
Inner membrane permeabilization induced by melittin and Trp-to-Ala-substituted Tritrp1 analogs. Peptide concentrations were MIC (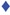), ^1^/_2_ MIC (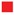), ^1^/_4_ MIC (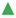), ^1^/_8_ MIC (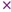), ^1^/_16_ MIC (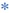), ^1^/_32_ MIC (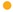) and 0 µM (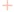). The MIC values for each peptide are derived from Table 1. For peptides with a range of MICs, the higher concentration was used. For A-Tritrp, the highest peptide concentration selected was 128 µM. The results are the average of three independent experiments, and the standard deviation (SD) for two selected peptide concentrations are depicted in Figure 10.

The use of membrane mimetic systems, such as LUVs, allows for the creation of well-defined lipid mixtures, but these do not represent all aspects of the highly complex and heterogeneous cytoplasmic bacterial membrane. Additionally, the peptide-to-lipid ratios used in the calcein leakage experiments might not fully represent a biological scenario. Despite the strong correlation previously described for ePC:ePG membrane permeabilization and the antimicrobial activity of Tritrp1 and its analogs, we felt that it was necessary to evaluate the ability to permeabilize an actual cytoplasmic bacterial membrane. To achieve this, we used the *E. coli ML35p* strain and the impermeable substrate ONPG (2-nitrophenyl-β-D-galactopyranose) as an indicator of membrane perturbation [49]. The effects induced by Tritrp1 and its Trp-substituted analogs on the *E. coli* inner membrane permeability are depicted in Figure 8 and Figure 9.

In these assays, the permeabilization of the *E. coli* inner membrane results in an increase of the A_420_ due to the hydrolysis of ONPG by β-galactosidase, an enzyme that is located in the cytoplasm [49]. An increase in the absorbance was detected over 60 min for most peptides in this study, indicating that the membranes were permeabilized by the peptides. At concentrations close to their MICs, the peptides seem to trigger membrane permeabilization after 20 min of incubation. At concentrations less than a quarter of the MIC, there is generally little membrane permeabilization caused by the peptides. In comparison to Tritrp1 (Figure 8), the Ala-derived peptides exhibited a higher level of membrane permeabilization at the MIC, indicating a strong tendency of these peptides to disturb the permeability of the inner membrane (Figure 9). However, it is important to consider that the MIC for most Ala-derived peptides is considerably higher than Tritrp1. The Tyr-derived peptides exhibited membrane permeabilization profiles similar to Tritrp1 at their MICs (Figure 8). However, compared with the well-known cytotoxic and membrane-active melittin peptide [50,51] (Figure 9), the membrane perturbing activity of Tritrp1 and its analogs is not as strong and takes a longer time to build up. This indicates that distinct mechanisms of action may be involved in the antimicrobial activity of these peptides.

**Figure 10:**
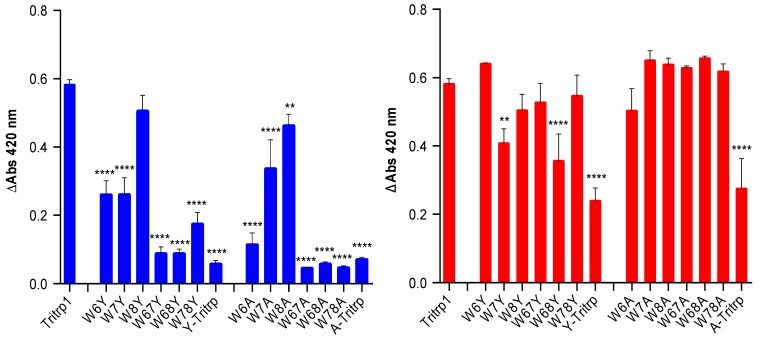
Change in absorbance (ΔAbs _420_) after 55 min of incubation of *E. coli* ML35p in the presence of Tritrp1 and its analogs at 4 µM (blue) and at the respective MICs for each peptide (red). For peptides with a range of MICs, the higher concentration was considered. Results are the mean ± SD (*n* = 3).

In order to identify the effect of the individual Trp substitutions on the membrane activity among all of the peptides in this study, the levels of permeabilization, represented by the changes in A_420_ after 55 min of incubation are depicted in Figure 10. Only two concentrations were selected, the MIC of the peptide and 4 µM, the latter being used to compare across all Tritrp1 analogs.

At their MICs, most of the Tyr- and Ala-derived peptides induce a similar level of ONPG hydrolysis compared to Tritrp1, which strongly suggests that their antimicrobial activity involves a membrane perturbing mechanism of action (Figure 10, red). However, some peptides (W7Y, W68Y and Y-Tritrp) show statistically significant lower membrane activity in comparison to Tritrp1 at their respective MICs (Figure 10, red). These results indicate that other mechanism(s) of action may contribute to the bactericidal activity of these peptides. Interestingly, A-Tritrp did not have a strong permeabilization ability for the *E. coli* inner membrane at 128 µM, which correlates with its lack of antimicrobial activity, despite causing significant effects against the ePC:ePG vesicles in the calcein leakage assays (Figure 4).

Using a common concentration of 4 µM to compare across all peptides, a trend emerged depending on the position of the Trp substitution (Figure 10, blue). Modifications of Trp6 substantially reduced the ability of the peptide to permeabilize the bacterial membrane. W6A and W6Y had a statistically significant lower permeabilizing capability in comparison to Tritrp1. Similarly W7A and W7Y also exhibited a considerably lower ability to permeabilize the inner membrane of *E. coli* (Figure 10, blue). In contrast, the permeabilizing ability of W8Y was not significantly affected (Figure 10, blue), suggesting that Trp8 is not as important for the activity of the peptide. These results indicate that the differences in the ability to permeabilize bacterial membranes are responsible for the differences in antimicrobial activity observed for the Tritrp1 analogs (Table 1). These results are in agreement with the position-dependent effects already described in the calcein leakage experiments (Figure 5).

The case of Y-Tritrp is interesting due to the statistically significant lower membrane permeabilization observed in *E. coli*. The peptide also exhibited an MIC of 16 µM (Table 1) and a Tritrp1 comparable membrane disturbing ability in ePC:ePG vesicles (Figure 4 and Figure 5). It is feasible that Y-Tritrp exerts its antimicrobial effect though a different mechanism, which, in combination with membrane permeabilization, might lead to cell death. A similar behavior was described for a Tritrp1 peptide analog, where all Trp residues were substituted by 5-hydroxy-Trp [24]. This peptide lacked the ability to permeabilize synthetic and bacterial membranes, but preserved a strong antimicrobial activity against *E. coli*. Its mechanism of action is not completely understood yet, but was related to either outer membrane destabilization or binding intracellular targets, which would induce the inhibition of macromolecular synthesis [24].

The significantly lower ability of A-Tritrp to permeabilize the bacterial inner membrane (Figure 10) could be correlated with the lack of antimicrobial activity against *E. coli* (Table 1). Considering the high percentage of calcein leakage observed previously for A-Tritrp (Figure 5), this work suggests that permeabilization of the inner membrane of *E. coli* correlates better than calcein leakage to the antimicrobial activity.

Altogether, our experiments show that the three Trp residues located at the core of tritrpticin do not make the same contribution to the antimicrobial activity. We know from previous work that Trp position is important for the cytotoxic activity of α-helical peptides [52]. Similarly, the antimicrobial activity of lactoferricin-derived peptides was strongly dependent on its Trp residues [19]. Accordingly, in the case of Tritrp1, the Trp residues in each of the three positions contribute differently to the antimicrobial activity of the peptide, as well as to the mechanism(s) of action that may be involved in causing bacterial death. The Trp residue located at position 6 was found to be the most important Trp residue for permeabilization *in vitro* and *in vivo*. Furthermore, this Trp proved to be crucial for the antimicrobial activity of Tritrp1. On the other hand, the Trp at position 8 did not have a substantial influence on the antimicrobial activity and membrane permeabilization properties of Tritrp1.

### 2.7. QSAR Analysis

An evaluation of the relative importance of the three Trp residues on the antimicrobial activity of tritrpticin was also attempted *in silico*. The use of a variety of amino acid descriptors for the quantitative structure-activity relationships (QSAR) analysis allows for the correlation of amino acids in a particular antimicrobial sequence to its biological potency [53,54]. A theoretical model was developed for the 15-amino acid residue bovine lactoferricin (LFB) peptide (FKCRRWQWRMKKLGA). This multivariate model, in addition to the native sequence of LFB, was developed on the basis of experimental results obtained for several substituted variants of the peptide, including substitution of its two Trp residues (for details, see [53,54,55]). The QSAR model was able to confirm that the second Trp was the most important amino acid residue for the antimicrobial activity of the LFB peptide [53]. When the same model was used to evaluate tritrpticin, Trp8 and, to a lesser degree, Trp7 were identified as the most relevant Trp residues for its antimicrobial activity. Unfortunately, this result did not coincide with our experimental data, where Trp6 was clearly shown to be more relevant for the antimicrobial activity against *E. coli*. This indicates that factors other than the three Trp residues also have a large effect on the antimicrobial activity of tritrpticin. Indeed Tritrp1 adopts a distinct three-dimensional micelle-bound structure when compared to the 15-residue LFB peptides [22,35,56]. In order to validate this theoretical approach further, it might be interesting in the future to develop a new model based on our data reported here for Tritrp1. This would allow us to determine whether the new model can be useful to predict available data for related Trp-rich peptides, such as indolicidin and puroindolines [25,57,58,59,60].

## 3. Experimental Section

### 3.1. Materials, Peptides and Bacterial Strains

Phospholipids and cholesterol were purchased from Avanti Polar Lipids (Alabaster, AL, USA). SDS-d_25_ was obtained from Cambridge Isotopes Laboratories (Andover, MA, USA). The peptides were purchased from GenScript Inc. (Piscataway, NJ, USA). They were synthesized using standard 9-fluorenylmethyoxycarbonyl (Fmoc) chemistry, and their identities and purities (>95%) were validated by mass spectrometry and HPLC, respectively. Melittin purified from honey bee venom and other chemicals were purchased from Sigma-Aldrich (St. Louis, MO, USA).

*E. coli* ATCC 25922 was purchased from American Type Culture Collection (Manassas, VA, USA). *E. coli* ML35p was kindly provided by Dr. Robert Lehrer at the UCLA David Geffen School of Medicine.

### 3.2. Antibacterial Activity

The minimum inhibitory concentration (MIC) of Tritrp1 and its Trp-substituted analogs against *E. coli* ATCC 25922 was measured by the standard broth microdilution method [61]. Bacteria (5 × 10^5^ CFU/mL) were incubated overnight at 37 °C in Mueller-Hinton broth (MHB) and in the presence of peptides diluted in a two-fold concentration series ranging from 0.25–128 µM in a 96-well polypropylene plate. The reported MIC values correspond to the minimum peptide concentration where bacterial growth was not observed. After overnight incubation, 10 µL from the first three wells without bacterial growth for each peptide in the MIC plate were diluted 1:10^6^ in MHB, and 100 µL of this dilution was plated in MHB-agar. After incubation overnight at 37 °C, the minimum bactericidal concentration (MBC) was reported as the minimum peptide concentration where no colony formation was detected.

### 3.3. ^1^H Nuclear Magnetic Resonance (NMR) Spectroscopy

The interaction of Tritrp1 and its Trp-substituted peptides with SDS micelles was studied by one-dimensional (1D) ^1^H NMR spectroscopy. The peptides (0.2 mM) were dissolved in 9:1 H_2_O:D_2_O, and the NMR spectra were acquired at 310 K in a Bruker Avance 700 MHz Ultrashield NMR spectrometer equipped with a Cryo-Probe (Bruker Corporation, Milton, ON, USA), using 2,2-dimethyl-2-silapentane-5-sulfonic acid (DSS) as the internal chemical reference. NMR spectra were then acquired for the peptide samples dissolved in 30 mM SDS-d_25_ micelles. The pH of all of the NMR samples was between 3.5 and 4.5.

### 3.4. Large Unilamellar Vesicles (LUVs) Preparation

LUVs were prepared by adding the necessary volume of egg-yolk phosphatidylcholine (ePC), egg-yolk phosphatidylglycerol (ePG) and cholesterol dissolved in chloroform. Two LUV systems were prepared: ePC:ePG (1:1) and ePC:cholesterol (2.5:1). The organic solvent was initially removed by evaporation in a stream of nitrogen gas and the remaining solvent was evaporated under vacuum overnight. The lipid films were resuspended in Tris-buffer (10 mM Tris, 150 mM NaCl, 1 mM EDTA, pH 7.4) by vigorous vortexing and then freeze-thawed five times using liquid nitrogen and lukewarm water. LUVs of 100 nm in diameter were produced by extrusion through two 0.1-µm polycarbonate filters (Nucleopore Filtration Products, Pleasanton, CA, USA). Calcein-containing LUVs were prepared by resuspending the dry lipids in Tris-buffer containing 70 mM calcein. After LUV extrusion, free calcein was removed by gel filtration using a Sephadex G-50 column. The concentration of lipids in the LUVs was measured by the Ames phosphate assay [62], performed in triplicate.

### 3.5. Tryptophan Fluorescence and Acrylamide Quenching

The tryptophan fluorescence was monitored using a Varian Cary Eclipse fluorimeter (Agilent Technologies, Santa Clara, CA, USA) equipped with a multicell sample holder and temperature control set to 37 °C. The peptides (1 µM) in Tris-buffer were excited at 280 nm (slit width: 5 nm) and the emission spectra were measured from 300 to 500 nm (slit width: 10 nm) in the absence and presence of LUVs (30 µM). The difference in the maximum emission wavelength between the peptide in buffer and in the presence of LUVs is taken as the blue shift.

For the acrylamide quenching experiments, sequential additions of 5 µL of 4 M acrylamide stock solution (up to 0.15 M) were made to the sample containing peptide (1 µM) and LUVs (30 µM). The tryptophan fluorescence was recorded after each acrylamide addition. The fluorescence intensity changes were analyzed through Stern-Volmer plots, where the quenching constants (Ksv) were calculated using the Equation:

$$\frac{Fo}{F}=1+Ksv[Q]$$

where *Fo* is the initial fluorescence of the peptide and *F* is the fluorescence at the acrylamide quencher concentration [*Q*].

### 3.6. Calcein Leakage

The ability of Tritrp1 and its analogs to permeabilize the phospholipid bilayer was measured by following the leakage of calcein induced in calcein-loaded LUVs. The calcein-loaded LUVs (2.5 µM) were incubated in the presence of different concentration of peptides (0–0.25 µM) in a 96-well plate and incubated for 15 min at 37 °C with constant shaking. The calcein fluorescence was measured by excitation at 485 nm and emission at 520 nm in an Eppendorf PlateReader AF2200 (Eppendorf, Mississauga, ON, Canada). The fluorescence of calcein-loaded LUVs in the absence of peptide was subtracted from all values, and Triton X-100 (0.1%) was used to establish the fluorescence intensity of 100% leakage. Results were calculated as the percentage of maximum leakage.

### 3.7. E. coli Inner Membrane Permeabilization

The permeabilization of the bacterial inner membrane induced by Tritrp1 and its Trp-substituted analogs was measured as described by Epand *et al.* [49]. This method uses the *E. coli* strain ML35p, which constitutively expresses the cytoplasmic β-galactosidase enzyme and lacks the *lac* permease transporter. In the presence of membrane permeabilizing agents, the membrane impermeable substrate 2-nitrophenyl-β-d-galactopyranoside (ONPG) is hydrolyzed by β-galactosidase, and the formation of its product can be monitored by absorbance at 420 nm.

*E. coli* ML35p was grown at 37 °C in Luria-Bertani (LB) broth from a single colony until OD_600_ ~0.6. The cells were collected and washed three times with incubation buffer (10 mM Na^+^-phosphate, pH 7.4, 100 mM NaCl and 300 µg/mL LB) and added to a final OD_600_ of 0.3 in the presence of 0.5 mM ONPG and two-fold peptide concentration series in a 96-well plate. The absorbance at 420 nm of the wells was measured every 2 min approximately for 60 min using a Perkin Elmer Victor™ X4 multi-label plate reader (Waltham, MA, USA) with shaking and temperature control at 37 °C.

### 3.8. Statistical Analysis

Statistical analysis was performed using GraphPad Prism 6.0 [63]. Statistical significant differences between the Tritrp1 analogs and the control peptide (Tritrp1) were established by using one-way ANOVA and Dunnett’s *post hoc* test. The *p*-value scale is as follows: *p* ≤ 0.05 (*), *p* ≤ 0.01 (**), *p* ≤ 0.001 (***) and *p* ≤ 0.0001 (****).

## 4. Conclusions

The position of the Trp residues in tritrpticin has a very important role in the membrane perturbing ability of this peptide. Not all residues were equally relevant for the antimicrobial mechanism of action. Our studies of the permeabilization of synthetic membranes established that Trp6 was the most important Trp residue for membrane disruption in contrast to Trp8. The same phenomenon was observed in a more biologically relevant scenario using assays that measure the permeabilization of the *E. coli* inner membrane in intact bacteria. These membrane effects were directly correlated with the antimicrobial potency of Tritrp1 and its Trp-to-Tyr and Trp-to-Ala analogs. These results not only highlight the importance of the Trp residues for the antimicrobial activity of Tritrp1, but also were able to assign individual contributions to each Trp depending on its location in the peptide sequence. This work contributes to a better understanding of the role of Trp residues in the antimicrobial activity of tritrpticin.
